# Zebrafish: A See-Through Host and a Fluorescent Toolbox to Probe Host–Pathogen Interaction

**DOI:** 10.1371/journal.ppat.1002349

**Published:** 2012-01-05

**Authors:** David M. Tobin, Robin C. May, Robert T. Wheeler

**Affiliations:** 1 Department of Molecular Genetics and Microbiology, Center for Microbial Pathogenesis and Center for AIDS Research, Duke University, Durham, North Carolina, United States of America; 2 Molecular Pathology and School of Biosciences, University of Birmingham, Birmingham, United Kingdom; 3 Department of Molecular and Biomedical Sciences and Graduate School of Biomedical Sciences, University of Maine, Orono, Maine, United States of America; Duke University Medical Center, United States of America

## What Are the Advantages of This System Compared to Other Infection Models?

**Figure 1:**
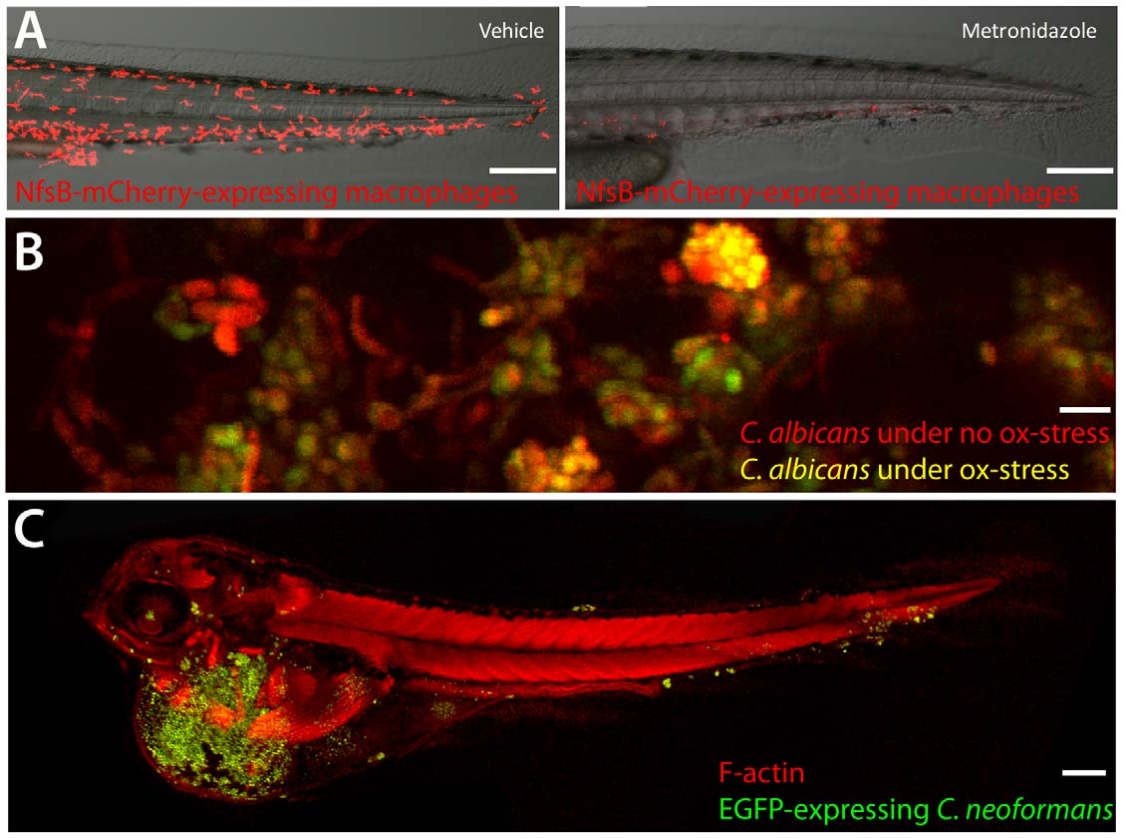
A sampling from the zebrafish toolbox. (A) Selective ablation of macrophages. Transgenic fish with macrophage-specific expression of Gal4 [2] and Gal4-dependent expression of nitroreductase-mCherry fusion protein were incubated at 3 dpf with 5 mM metronidazole or vehicle for 24 hours. Neither transgenics nor controls exposed to metronidazole had any loss of viability or developmental defects. Ablation efficiency of macrophages is >90% (R. Gratacap and R. Wheeler,unpublished data). Scale bar = 100 µm. (B) OXYellow *Candida albicans* reports on oxidative stress in vivo. Zebrafish larvae were infected in the hindbrain with OXYellow *C. albicans* (expressing mCherry constitutively and EGFP under the oxidative stress-induced catalase promoter) and imaged at 24 hours post-infection. Green/red ratio quantifies oxidative stress (K. Brothers and R. Wheeler, unpublished dat). Scale bar = 10 µm. (C) *Cryptococcus neoformans* infects zebrafish embryos. Zebrafish were infected with EGFP-expressing *C. neoformans* and imaged. Clusters of fungi are seen in the tail (S. Johnston and R. Ma, unpublished data). Scale bar = 100 µm.

In many ways, the zebrafish represents a hybrid between mouse and invertebrate infection models. Powerful forward-genetic tools that have made invertebrates justifiably famous are not only relatively accessible in the zebrafish, but have been exploited to yield new insights into human infectious diseases, including leprosy and tuberculosis [1]. Transgenic technologies have enabled detailed, non-invasive in vivo visualization of macrophages and neutrophils in pitched battle with bacteria and fungi [2], [3]. Reverse genetics with morpholinos, vivo-morpholinos, and zinc-finger nucleases (but unfortunately not homologous recombination, which for the moment remains out of reach in this organism) enable examination of the roles of specific genes during infection. Flexible genetic systems such as Gal4-UAS and Cre-Lox permit tissue-specific transformation and ablation ([3]; Figure 1).

These technologies can be applied to hundreds of embryos in a single day. Zebrafish embryos at the one- to four-cell stage are microinjected with morpholinos to target translation or splicing of specific transcripts, or to limit microRN (mRNA) activity. This knockdown can be effective for up to 10 days post-fertilization, allowing relatively long-term imaging of infection in the background of specific gene knockdowns. Similarly, early microinjection with mRNA for the Tol2 transposase along with DNA constructs bracketed by Tol2 repeats results in remarkably efficient transgenesis. From injection to the establishment of a stable transgenic line can be less than eight weeks.

## Is the Zebrafish Immune System Similar to the Human?

The short answer is yes, very similar. We share a similar developmental program, a comparable set of specialized immune cells including B and T cells, and a similar suite of immune signaling molecules. Recent studies on the monocytic phagocyte system, dendritic cells, and eosinophils show that the more we study the zebrafish immune system, the more similarities we find. Although zebrafish have both innate and adaptive arms of immunity, as in mammals, the adaptive arm takes longer to develop, and therefore innate immunity is the sole protector of young fish up to 4 weeks old. Thus, initial host–pathogen interactions can be studied in isolation in the zebrafish larva. There are some important differences, particularly in the adaptive immune response where sites of maturation differ and there are distinctIg subtypes[4], [5]. Nevertheless, zebrafish are naturally infected by many of the same classes of pathogens that affect mammals. Thus, fundamentally conserved frameworks of host–pathogen interactions can be studied in a facile model.

## How Can the Transparency and Small Size of Zebrafish Be Exploited?

The most impressive feature of this model is the ability to perform non-invasive, high-resolution, long-term time-lapse and time-course experiments to visualize infection dynamics with fluorescent markers. This sets zebrafish apart from both in vitro and mammalian in vivo infection models, as summarized in Table 1. A variety of genetically encoded probes, fluorescent physiological indicator chemicals, cell type–specific fluorescent transgenes, photoactivatable proteins, and pathogen-encoded conditional reporters (for example, indicating oxidative stress or phagocytosis; Figure 1) has lit up mechanisms of bacterial, fungal, and viral pathogenesis. A particularly elegant use of the see-through fish is to photoactivate fluorescent proteins [2], prodrugs (Cre-ER; [6]), or “killer” proteins(KillerRed; [7]) to spatially restrict the desired effect. The transparency of wild-type larvae and casper mutant adults [8] provides a unique portal for observing and testing the impact of molecular perturbation on true infection dynamics in the intact host.

**Table 1:** Advantages of embryonic zebrafish model for study of innate immune-pathogen interaction.

Limitations of In Vitro Phagocyte Challenge	Advantages of Larval Zebrafish Model
Purification of immune cells can perturb function	Purification unnecessary
Media does not recapitulate tissue-specific in vivo nutrients	In vivo nutrients
No soluble factors (e.g., opsonins, cytokines) from other cell types	Normal soluble components
No contact activation or inhibition by other cell types	Normal tissue environment
No effect of extracellular matrix interactions	Normal extracellular environment
Cannot monitor dissemination of infection	Tissue-to-tissue dissemination can be imaged

Limitations of In Vivo Mouse Infection	Advantages of Larval Zebrafish Model
Too large to examine infection host-wide at high resolution	Possible to image entire live fish
Opaque skin and organs limit fluorescent imaging below ∼100 µm	Fish larvae are transparent
Elimination of macrophage function pleiotropic	Temporary macrophage ablation feasible
Very limited high-resolution, non-invasive imaging of pathogen or immune morphology	High-resolution, non-invasive imaging facile throughout the host

The large clutch size and the unusual ability to create gynogenetic diploids has allowed the first forward genetic screen to identify vertebrate host determinants of immunity to mycobacterial infection [1]. Other recent work demonstrates the utility of high-throughput screening to identify mycobacterial mutants with altered virulence [9], [10], whilst recent advances in automated screening now enable high-content screening of embryos [11], [12]. Embryos and young larvae are relatively permeable to small molecules, and the zebrafish embryo is small enough to develop in a well of a 384-well plate. High-throughput chemical genetic screens are made easier by direct introduction of chemicals into the water, and can be applied to identify novel antimicrobial drugs [13].

Another remarkable opportunity of this small transparent model comes from its complex anatomy, which enables infection through multiple routes of infection in an intact host with a complex immune system. Thus, fish viruses can be inoculated through immersion or microinjection, mycobacterial infection can be modeled by localized hindbrain injection or direct injection into the bloodstream, and pseudomonad interaction can be examined in the gastrointestinal tract as well as in the hindbrain and through intravenous injection. This versatility emphasizes the unique position of this model for understanding infection dynamics.

## What Limits Use of the Zebrafish to Model Infection, and How Can These Limits Be Turned into Advantages?

The use of any model host necessitates a trade-off in order to ask new experimental questions. For instance, there are some important anatomical differences between zebrafish and mammals (gills instead of lungs, hematopoesis in the anterior kidney instead of bone marrow, lack of discernable lymph nodes, and a very different reproductive system) that constrain the range of infections that can be successfully studied in the zebrafish. In comparison to traditional model systems for pathogenesis, most notably the mouse, there is a lack of antibody reagents available. Antibodies raised against well-conserved mammalian proteins often demonstrate cross-reactivity with zebrafish orthologs, and there are concerted efforts in the zebrafish community to increase the number of antibodies raised specifically against zebrafish proteins. Nonetheless, this remains a current limitation of the model. The zebrafish larva grows well at water temperatures between 22°C and 33°C and lacks adaptive immunity until approximately 1 month post-fertilization. Thus, the zebrafish is well-suited to the study of cold-adapted or broad host-range pathogens [1], whilst on the positive side the ability to rear fish at different temperatures allows manipulation of infection that is not possible with other vertebrate model hosts [14]. The natural lack of adaptive immunity early in development limits the possibility of examining innate-adaptive crosstalk in the transparent embryo. But on the other hand, this developmental feature has permitted an unprecedented elucidation of innate immune functions that regulate immunity to *Mycobacterium marinum*, a fish pathogen closely related to the global human pathogen *Mycobacterium tuberculosis*. Furthermore, if adaptive immune function is to be tested, transparent “casper” adult fish can be used to image fluorescent events non-invasively [8]. As a general rule, zebrafish are also more tolerant of serious abnormalities than mammalian models (for instance, animals with essentially no cardiac function are viable for a few days after hatching), providing a unique opportunity to study mutants that are not available in rodent models [15].

## What Are Unexpected Findings Pioneered Using the Zebrafish System and Validated in Mammals?

The unique power of the zebrafish model has led to several breakthroughs in our understanding of infectious disease. Studies of *M. marinum*, in particular, have yielded novel insight into the role of specific eicosanoids in host defense [1], the role of macrophages in promoting pathogen dissemination [16], infection-induced antibiotic tolerance [17], and the role of the ESX secretion system in granuloma formation [18]. In the case of mycobacteria, conserved virulence mechanisms and host susceptibility determinants identified during zebrafish infection have been validated in *M. tuberculosis* and human susceptibility. Zebrafish are now being used to model infections as disparate as *Leptospira* and *Cryptococcus* (Figure 1). As new models progress past the methodology phase, we are starting to gain real-time insight into host–pathogen interactions as varied as viral-induced hemorrhaging [14], CFTR-dependent immune responses to bacteria [19], and NADPH oxidase-mediated control of fungal filamentation [20]. These, and many more studies than could be mentioned here, should shed new light on a broad range of host–pathogen interactions driving human infectious diseases.
